# This Article Corrects: “A Chemist with a Strange Etiology of Rhabdomyolysis: A Case Report of a Rare Toxicological Emergency”

**DOI:** 10.5811/cpcem.2022.2.56452

**Published:** 2022-02-13

**Authors:** Rajadurai Meenakshisundaram, Joshua Vijay Joseph, Prabakaran Perumal, Akmal Areeb, Prathap Pancheti, Dinesh Kannan Sampath, Esther Monica Jared, Allison K Ryan

**Affiliations:** * Apollo KH Hospital, Department of Emergency Medicine and Critical Care, Melvisharam, Tamil Nadu, India; † Einstein Medical Center, Department of Emergency Medicine, Philadelphia, Pennsylvania

*Clin Pract Cases Emerg Med. 2021:5(4)432–435*.

A Chemist with a Strange Etiology of Rhabdomyolysis: A Case Report of a Rare Toxicological Emergency

R Meenakshisundaram, JVJoseph, P Perumal, A Areeb, P Pancheti, DK Sampath, EM Jared, AK Ryan

[Clin Pract Cases Emerg Med. 2022;6(1):99.] This article includes an additional author, Allison K Ryan, MD.

